# IsoSeq transcriptome assembly of C_3_ panicoid grasses provides tools to study evolutionary change in the Panicoideae

**DOI:** 10.1002/pld3.203

**Published:** 2020-02-28

**Authors:** Daniel S. Carvalho, Aime V. Nishimwe, James C. Schnable

**Affiliations:** ^1^ Department of Agronomy and Horticulture Center for Plant Science Innovation University of Nebraska‐Lincoln Lincoln NE USA

**Keywords:** C_4_ photosynthesis, grasses, panicoideae, phylogenetics, transcriptomics

## Abstract

The number of plant species with genomic and transcriptomic data has been increasing rapidly. The grasses—Poaceae—have been well represented among species with published reference genomes. However, as a result the genomes of wild grasses are less frequently targeted by sequencing efforts. Sequence data from wild relatives of crop species in the grasses can aid the study of domestication, gene discovery for breeding and crop improvement, and improve our understanding of the evolution of C_4_ photosynthesis. Here, we used long‐read sequencing technology to characterize the transcriptomes of three C_3_ panicoid grass species: *Dichanthelium oligosanthes*, *Chasmanthium laxum*, and *Hymenachne amplexicaulis*. Based on alignments to the sorghum genome, we estimate that assembled consensus transcripts from each species capture between 54.2% and 65.7% of the conserved syntenic gene space in grasses. Genes co‐opted into C_4_ were also well represented in this dataset, despite concerns that because these genes might play roles unrelated to photosynthesis in the target species, they would be expressed at low levels and missed by transcript‐based sequencing. A combined analysis using syntenic orthologous genes from grasses with published reference genomes and consensus long‐read sequences from these wild species was consistent with previously published phylogenies. It is hoped that these data, targeting underrepresented classes of species within the PACMAD grasses—wild species and species utilizing C_3_ photosynthesis—will aid in future studies of domestication and C_4_ evolution by decreasing the evolutionary distance between C_4_ and C_3_ species within this clade, enabling more accurate comparisons associated with evolution of the C_4_ pathway.

## 1 INTRODUCTION

The pace of plant genome sequencing has accelerated in recent years. However despite decreases in sequencing costs and improvements in genome assembly quality, species selected for whole genome sequencing often meet one or more of the following criteria: (a) agricultural importance, (b) status as a genetic model system, or (c) ecological importance. Sequence data from species which lack direct economic, ecological, or genetic model importance can enable comparative analyses to address biological questions in crops and model species (Ellegren, 2014; Michael & Jackson, 2013). C_4_ photosynthesis has evolved multiple times in the grasses (GPWG II, 2012), making it particularly amenable to study through comparative genetic approaches (Huang, Studer, Schnable, Kellogg, & Brutnell, 2016; Wang et al., 2009). C_4_ photosynthesis requires both substantial biochemical and anatomical changes (Kellogg, 2013). All grasses which utilize the C_4_ pathway belong to the PACMAD clade, a group of grass subfamilies and tribes which includes substantial numbers of both C_3_ and C_4_ species (GPWG II, 2012). Substantial new insights into both the genes involved in producing the biochemical and anatomical changes required for C_4_ photosynthesis, as well as the potential function of individual amino acid residues can be obtained from comparative analysis of individual gene families across species utilizing either C_3_ or C_4_ photosynthesis within the PACMAD clade (Christin, Arakaki, Osborne, & Edwards, 2015; Christin, Salamin, Savolainen, Duvall, & Besnard, 2007; Moreno‐Villena, Dunning, Osborne, & Christin, 2017). However, assembling sequence data for a single gene family from a large enough set of species through PCR amplification and individual Sanger sequencing remains a time‐ and labor‐intensive process.

Many domesticated grasses belong to the PACMAD clade, including maize (*Zea mays*), sugarcane (*Saccharum *spp.), sorghum (*Sorghum bicolor*), and foxtail millet (*Setaria italica*). However, every domesticated grass in the PACMAD clade with a sequenced genome utilizes one or more variants of the C_4_ photosynthetic pathway (Bennetzen et al., 2012; Schnable et al., 2009). As a result, while published whole genome sequence assemblies exist for at least 14 grasses within the PACMAD clade (Table 1), only one of these (*Dichanthelium oligosanthes*, a wild species) (Studer et al., 2016) utilizes C_3_ photosynthesis. Long‐read sequencing can effectively generate sequence for large numbers of full‐length cDNAs even in species lacking reference genome assemblies (An, Cao, Li, Humbeck, & Wang, 2018; Zhang et al., 2019). One concern with utilizing this technology for comparative genetic studies is that the higher error rate, particularly the frequencies of insertion and deletion errors, makes data from long‐read‐based sequencing of non‐model species unsuitable for use in comparative evolutionary analyses (Gonzalez‐Garay, 2016). However, we previously found that observed synonymous substitution rates calculated from consensus sequences constructed using PacBio IsoSeq pipeline were not elevated relative to a sister lineage where gene sequences were taken from a sanger‐based whole genome assembly, indicating sequence data obtained in this manner may indeed be suitable for comparative evolutionary analyses (Yan et al., 2019).

**Table 1:** Published reference genomes for grass species within the PACMAD clade

Species	Relevance	C_3_/C_4_	Genome publication
*Dichanthelium oligosanthes*	Wild Species	C_3_	Studer et al. (2016)
*Eleusine coracana* ^a^	Grain Crop	C_4_	Hittalmani et al.(2017)
*Eragrostis tef* ^a^	Grain Crop	C_4_	Cannarozzi et al. (2014) VanBuren et al. (2019)
*Miscanthus x giganteus* ^b^	Biomass Crop	C_4_	Swaminathan et al.(2010)
*Oropetium thomaeum* ^a^	Wild Species	C_4_	VanBuren et al. (2015), VanBuren et al. (2018)
*Panicum hallii* ^c^	Wild Species	C_4_	Lovell et al. (2018)
*Panicum miliaceum* ^c^	Grain Crop	C_4_	Zou et al. (2019)
*Panicum virgatum* ^c^	Biomass Crop	C_4_	Casler et al. (2011)
*Pennisetum glaucum* ^c^	Grain Crop	C_4_	Varshney, Shi, et al. (2017)
*Saccharum *spp.^b^	Sugar Crop	C_4_	Garsmeur et al. (2018)
*Setaria italica* ^c^	Grain Crop	C_4_	Bennetzen et al. (2012)
*Setaria viridis* ^c^	Genetic Model	C_4_	Brutnell et al. (2010)
*Sorghum bicolor* ^b^	Grain/Biomass/Sugar Crop	C_4_	Paterson et al. (2009)
*Zea mays* ^b^	Grain Crop & Genetic Model	C_4_	Schnable et al. (2009)

Note

Species sharing a common inferred evolutionary origin of C_4_ photosynthesis as reported in (GPWG II, 2012) are indicated by superscript letters.

**Figure 1:**
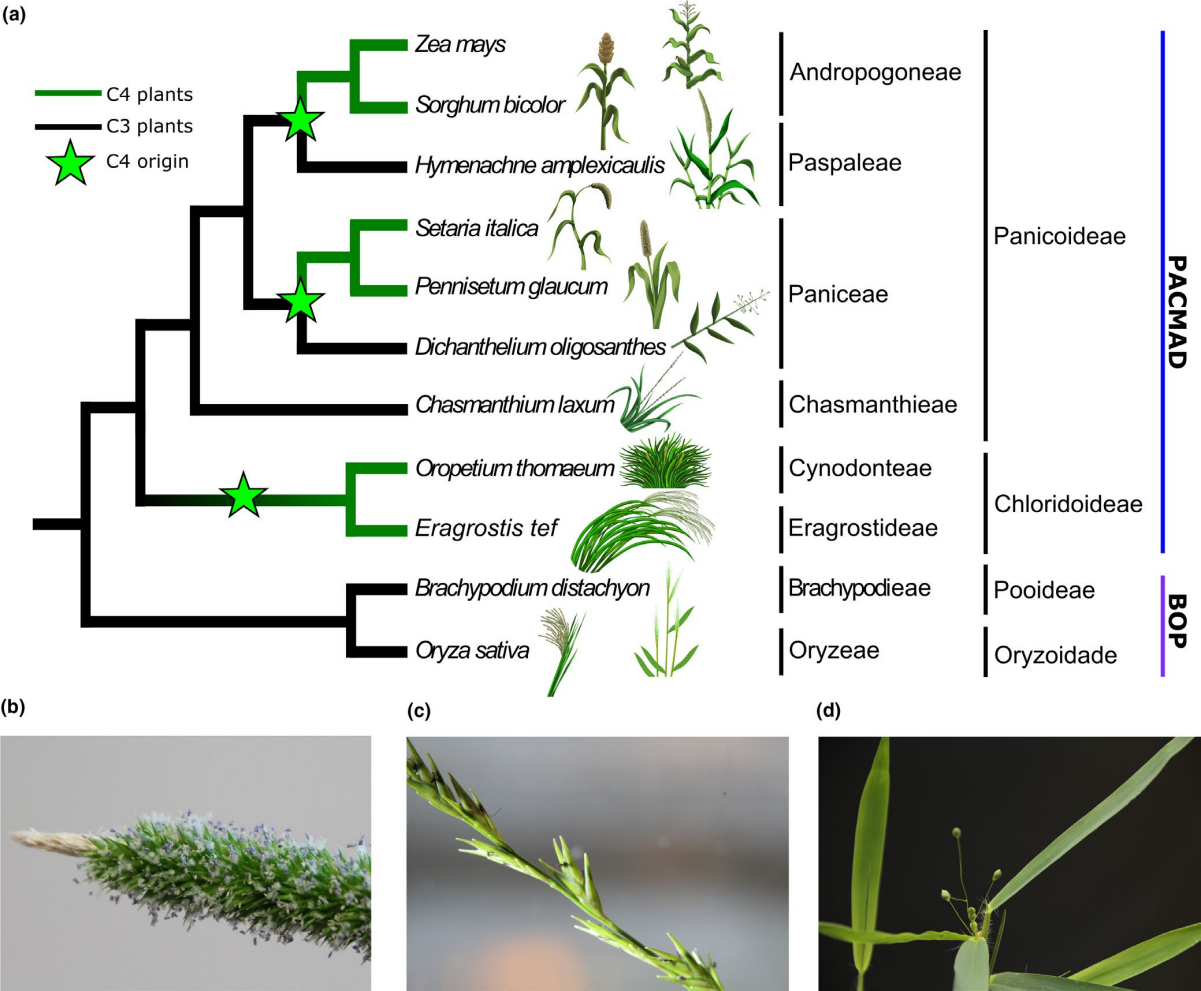
(a) Current literature consensus phylogeny of the relationships between the grass species studied here. Lineages in green utilize C_4_ photosynthesis, while lineages in black utilize C_3_ photosynthesis. The green stars indicate apparent independent origins of C_4_ photosynthesis. (b) Inflorescence of *Hymenachne amplexicaulis*. (c) Inflorescence of *Chasmanthium laxum*. (d) Inflorescence of *Dichanthelium oligosanthes*

Here, we report the sequencing and characterization of IsoSeq‐based transcriptomes for three additional PACMAD grasses, selecting to enable wider scale studies of protein sequence changes associated with the many parallel origins of C_4_ photosynthesis within that clade (Figure 1). These species were specifically selected to augment C_3_/C_4_ comparisons: *Hymenachne amplexicaulis*, *Chasmanthium laxum*, and *D. oligosanthes*. *H. amplexicaulis* is a member of the grass tribe Paspaleae which contains a mixture of C_3_ and C_4_ species. The Paspaleae are sister to exclusively C_4_ clade. This C_4_ clade is variously considered to either consider of two tribes the Andropogoneae and Arundinelleae or a single expanded Andropogoneae including those species otherwise included in the Arundinelleae. In either nomenclature, this clade includes both maize and sorghum, two species with extensive genomic, genetic, and phenotypic resources. *H. amplexicaulis* is found in moist habitats and thrives under flooded conditions (Kibbler & Bahnisch, 1999). *Chasmanthium laxum* belongs to the grass tribe Chasmanthieae (7 species). The Chasmanthieae all appear to utilize C_3_ photosynthesis (Kellogg, 2015) and are generally placed as early diverging lineage within the Panicoideae, the grass sub‐family containing maize, sorghum, sugarcane, miscanthus, switchgrass, foxtail millet, and proso millet (GPWG II, 2012). *C. laxum* can occur in a variety of environments such as woods, meadows, and swamps (Yates, 1966). The final species targeted for transcriptome sequences was *Dichanthelium oligosanthes*. *D. oligosanthes* is the only PACMAD species exclusively utilizing C_3_ photosynthesis with a published genome sequence to date (Studer et al., 2016). It is a member of the grass tribe Paniceae, a group which also includes foxtail millet, proso millet, and switchgrass, but is an outgroup to the MPC C_4_ subclade of exclusively C_4_‐utilizing species within that tribe (Giussani, Cota‐Sánchez, Zuloaga, & Kellogg, 2001; GPWG II, 2012; Washburn et al., 2017; Washburn, Schnable, Davidse, & Pires, 2015). As the published *D. oligosanthes* reference genome was constructed utilizing short‐read sequencing, the inclusion of *D. oligosanthes* provided an opportunity to improve the proportion of genes with full‐length sequences from this lineage available for comparative analyses. *D. oligosanthes* is present in small glades on the edge of woods (A. J. Studer, personal communication, April 08, 2019). The placement of *C. laxum* as an outgroup to other panicoid grasses with sequenced reference genomes and *D. oligosanthes* as sister to other members of the Paniceae with sequences reference genomes were recovered in a preliminary analysis of our long‐read dataset. Support of the placement of *H. amplexicaulis* as a sister group to Andropogoneae (sorghum and maize) was strong but not unambiguous.

## 2 METHODS

### 2.1 Plant material, RNA extraction, and sequencing

For all three species, young leaf tissue was harvested from mature plants growing in the greenhouses of the University of Nebraska's Beadle Center, latitude: 40.8190, longitude: −96.6932, on October 05, 2017. Young leaves were harvested from a *C. laxum* plant germinated from seed collected with accession Kellogg 1268 in Corkwood Conservation Area, just outside of Neelyville, MO, USA. Full details of this collection are published on Tropicos: https://www.tropicos.org/Specimen/100877982. Leaf tissue from *D. oligosanthes* was harvested from a plant descended from Kellogg 1175, which was collected in Shaw Nature Reserve, west of St. Louis, MO, USA. Full details of this collection are published on Tropicos: http://www.tropicos.org/Specimen/100315254. The specific *D. oligosanthes* plant used as a tissue donor had experienced at least three generations of selfing relative to the originally collected plant. This selfing occurred via an independent lineage from the F2 plant derived from the same collection which was used to generate the DNA for the *D. oligosanthes* reference genome (Studer et al. (2016). Young leaves were harvested from *H. amplexicaulis* which had been clonally propagated from collection PH2016. PH2016 was originally collected by Pu Huang in Myakka River state park in Florida, USA, on March 22, 2016. A clone of this same accession, grown in the same greenhouse, is deposited at the University of Nebraska‐Lincoln Herbarium with index number NEB‐328848.

Tissue samples were ground in liquid N2, and then, approximately 200mg of powdered tissue was added to 2 μl of TriPure isolation reagent (Roche Life Science, catalog number #11667157001). The RNA samples mixed with TriPure were then separated using chloroform, precipitated using isopropanol, and RNA pellets were washed using 75% ethanol. The samples were air‐dried and diluted in RNAsecure (Ambion). Total RNA concentration was measured using a NanoDrop 1,000 spectrophotometer, and the integrity was assessed based on electrophoresis on a 1% agarose gel. 10 μl of total RNA for each species was shipped to the Duke Center for Genomic and Computational Biology (GCB), Duke University, USA. Concentrations at the time of shipment ranged from 226.07 to 1,374 ng/μl. OD260/280 ratios for RNA samples were as follows: 1.93 (*H. amplexicaulis*), 1.92 (*C. Laxum*), and 2.03 (*D. oligosanthes*) within the recommended range for IsoSeq library construction of 1.8–2.2 provided by PacBio. One IsoSeq library was constructed per species, and each library was sequenced using a single SMRT cell on a PacBio Sequel.

### 2.2 Consensus reads and transcriptome assembly

Two separate sequence datasets were produced per library: full‐length (FL) transcripts and non‐full‐length (NFL) transcripts. A given transcript was considered FL if the sequence read contained both 5' and 3' adapters as well as poly‐A tail and are not redundant to other transcripts. The transcripts lacking the poly‐A tail or one of the adapters are instead included in the non‐full‐length dataset. Sequence reads from both files were used to assemble consensus transcriptomes using the software pbtranscript to cluster redundant sequences, part of the SMRT pipe package (version 5.1) with default parameters (https://www.pacb.com/wp-content/uploads/SMRT_Tools_Reference_Guide_v600.pdf). For each final consensus transcript, the single longest ORF present within that transcript was selected as the CDS sequence for downstream analyses. Consensus transcripts were obtained from full‐length and non‐full‐length transcripts generated from oligo‐dT purified mRNAs. As oligo‐dT purification can capture the 3' ends of partially fragmented mRNA molecules, it was anticipated that some transcripts may be less than full‐length and missing start codons. Therefore, ORFs were required to include an in‐frame stop codon but were not required to include an in‐frame "ATG" which may result in additional non‐translated codons being appended to the 5' end of the putative CDS but avoids CDS truncation when the 5' end of the sequence was not recovered.

### 2.3 Sequence data from species with published reference genomes

CDS file containing only one primary transcript per gene downloaded from Phytozome 12 (https://phytozome.jgi.doe.gov/pz/portal.html) was used from *Brachypodium distachyon* (International Brachypodium Initiative, 2010), *Oryza sativa* (rice) (Kawahara et al., 2013; Yu et al., 2005), *Sorghum bicolor* (sorghum) (Paterson et al., 2009), and *Setaria italica* (foxtail millet) (Bennetzen et al., 2012). CDS sequences for version 2 of the *Oropetium thomaeum* (oropetium) genome (Genome ID 51527) (VanBuren, Wai, Keilwagen, & Pardo, 2018) and the draft *Eragrostis tef* genome (Genome ID 50954) (VanBuren et al., 2019) were downloaded from CoGe (Lyons & Freeling, 2008). CDS sequences for the initial release of the *Pennisetum glaucum* (pearl millet) genome were downloaded from GigaDB (Varshney, Liu, Shi, Vigouroux, & Xu, 2017; Varshney, Shi, et al., 2017). CDS sequences for B73_RefGenV4 of the *Zea mays* (maize) reference genome were retrieved from Ensembl (Jiao et al., 2017). In cases where only a complete set of CDS sequences was released for a given species, we arbitrarily selected the longest annotated transcript from a given locus to be the single representative transcript for downstream analyses. mRNA sequences from *D. oligosanthes* were obtained CoGe (Genome ID 35847) (Studer et al., 2016) for comparison with IsoSeq *D. oligosanthes* transcripts. PEPC and PPDK gene families from sorghum for further manual curation and phylogeny analysis were obtained from Christin et al., (2007) and Wang et al., (2009), respectively.

### 2.4 Putative orthology assignments

CDS sequences obtained from *H. amplexicaulis*, *C. laxum,* and *D. oligosanthes* as described above were compared to the primary CDS sequences of each annotated gene in the sorghum genome using LASTZ version 1.04.00 (Harris, 2007) with the following parameters: –identity = 70 –coverage = 50 –ambiguous = iupac, –notransition, and –seed = match12. CDS sequences from the three target species were presumed to belong to an orthologous group as a given sorghum gene if the sorghum CDS sequence and target species CDS sequence were reciprocally identified as each other’s high scoring hit in the LASTZ analysis. The comparison of D. oligosanthes consensus transcripts and annotated mRNA sequences from the published reference genome for *D. oligosanthes* defined equivalence as sequences which were reciprocally identified as highest scoring LASTZ matches.

Orthologous relationships between sorghum genes and genes in other species with sequenced reference genomes were inferred based on syntenic orthology. For each combination of sorghum and rice, brachypodium, oropetium, teff, foxtail millet, pearl millet, sorghum, and maize all‐by‐all LASTZ comparisons were performed using the same parameters described above. The resulting LASTZ output was employed to identify initial syntenic genomic blocks using QuotaAlign with the parameters –tandemNmax = 10, cscore = 0.5, –merge and –Dm = 20 (Tang et al., 2011). The quota was set to –quota = 1:2 for maize and teff, and –quota = 1:1 for all other species. Pairwise syntenic block data were merged and polished using the methodology previously described in (Zhang et al., 2017) to obtain the final set of high confidence syntenic ortholog groups employed for all downstream analysis.

Orthology was treated as a transitive property; thus, each *H. amplexicaulis*, *C. laxum,* or *D. oligosanthes* gene identified as putatively orthologous to a given sorghum gene based on reciprocal best LASTZ hit analysis was also considered to be putatively orthologous to syntenic orthologs of that sorghum gene identified in each of the other species described above. The final sets of putatively orthologous gene groups including both sequences from published reference genomes and the long‐read sequencing described here are provided in Supporting Information.

### 2.5 Sequence alignment, QC, and phylogenetic analysis

Kalign (v2.04) was used to create a multiple sequence alignment from protein sequences obtained by translating CDS sequences from all genes in a give putatively orthologous gene group. This gapped protein alignment was in turn employed to create a codon‐level DNA alignment of the original CDS sequences. GBlocks version 0.91 was run with default parameters to identify high‐quality portions of the sequence alignment and remove those portions of the alignment not meeting specified quality thresholds (Talavera & Castresana, 2007). Alignments including only those portions passing GBlocks filtering were then used as input for RAxML version 8, using the GTRGAMMA model and with a clade of rice and brachypodium specified as an outgroup, to obtain a phylogenetic tree for each group of putatively orthologous genes (Stamatakis, 2014). When RAxML was unable to construct a phylogeny in which rice and brachypodium formed monophyletic clade sister to other taxa, the trees were omitted from downstream visualization. To plot all phylogenies, we used Densitree, part of the BEAST2 package, was used to create combined plots of large numbers of trees (Bouckaert et al., 2014). We performed bootstrap analyses of 100 randomly chosen trees (from all trees generated without any filtering step) with 100 replicates to obtain the branch support values of the most common tree topologies of the Densitree plot. For the trees built for PPDK and PEPC, RAxML bootstrap analyses were performed with 1,000 replicates. For visualization purposes only, all branches were treated as having equal length in order to improve the ease of visually comparing differences in topology. Data on the consistency or inconsistency of individual portions of the phylogeny were judged from comparisons of the single best trees generated using the sequence of each separate gene. However, it should be noted that bootstrapping was not performed for the individual best trees for each gene; hence, some disagreements in topology may simply reflect poorly resolved nodes with limited support.

As a result of the separate whole genome duplications in the maize and teff lineages, in many cases gene and species trees would contain different numbers of leaf nodes. For gene groups where maize and teff had each fractionated back to single copy status, only a single alignment file was created. If fractionation had already occurred in one lineage, but not the other, two separate alignments were created, each sampling one of the two co‐orthologous gene copies from the species with a retained whole genome duplication‐derived gene pair. When fractionation had not occurred in either lineage, four total alignments were generated per gene group, capturing all possible pairwise combinations of the two teff gene copies and two maize gene copies.

## 3 RESULTS AND DISCUSSION

The number of raw reads generated per species was largely consistent and ranged from 708,681 to 734,932 (Table 2). After clustering both full‐length and non‐full‐length transcripts to obtain a set of polished consensus transcripts, the number of sequences per species dropped to 164,640 to 193,422 (Table 2). The average length of consensus sequences ranged from 925 bp to 1,438 kb (Figure S1). A comparison between the mRNA sequences and IsoSeq transcripts from *D. oligosanthes* was performed to assess the improvement of sequence coverage. Out of the 13,847 reciprocal best LASTZ hits between the mRNA and IsoSeq data, 12,347 transcripts were longer than the mRNA sequences, while the remaining 1,500 sequences were either shorter or the same length in both datasets (Figure S2). The longer sequences recovered in this experiment may be the result of a combination of fragmentation within the *D. oligosanthes* reference genome and improved capture of 5′ and 3′ untranslated regions, which are often missed by homology‐based annotation of genomic sequence. While *H. amplexicaulis* exhibited the shortest consensus transcript length, this was not reflected in a reduced number of complete ORFs—those containing both an in‐frame ATG and stop codon and occupying at least 60% of the total transcript length. The number of consensus transcripts significantly exceeded the expected number of expressed genes; however, this is consistent with other reference genome‐free IsoSeq analyses (Kuang, Sun, Wei, Li, & Sun, 2019; Li et al., 2017; Yan et al., 2019). Inflated numbers of consensus transcripts can result from sequencing of multiple alternatively spliced isoforms of the same gene, sequencing of incompletely processed mRNA molecules (Martin et al., 2014), high sequence error rates preventing multiple sequences from the same transcript being collapsed into a consensus, divergent haplotypes of the same locus present in our clonally propagated, wild collected, or partially inbred starting material, or contamination of the original samples with mRNA from non‐target organisms.

**Table 2:** Summary statistics for raw and processed long read sequence data generated from each of the three target species

Species	Total reads	Raw data	CCS reads	FL reads	Average FL length	Consensus transcripts	Average consensus transcript length	Transcripts containing start codon
*Hymenachne amplexicaulis*	734,932 reads	5.8 GB	732,158 reads	284,027 reads	963 bp	193,422	925 bp	34,016/193,422 (17.5%)
*Dichanthelium oligosanthes*	708,681 reads	10.1 GB	701,802 reads	380,381 reads	1,460 bp	190,632	1,438 bp	36,055/190,632 (18.9%)
*Chasmanthium laxum*	729,710 reads	12.5 GB	649,149 reads	306,566 reads	1,294 bp	164,640	1,236 bp	26,490/164,640 (16%)

Alignment of final consensus reads to the sorghum reference genome was employed to estimate coverage of the shared grass gene space for data collected from each of our target species, as well as to assist in further collapsing multiple redundant sequences originating from alternative splicing, incomplete processing, or divergent haplotypes of transcripts originating from a single genetic locus. In all three cases, the majority of consensus transcripts could be aligned to known genes in the sorghum genome, with an average of between 9.3 and 12.1 consensus transcripts aligning to each sorghum gene represented in the transcriptome data (Table 3). Each of these three target species is predicted to be diploid based on either flow cytometry‐based estimates of genome size or imaging of chromosomes; thus, a maximum of two transcripts per locus can be explained by divergent haplotypes. The high number of consensus sequences aligned per represented sorghum locus suggests that a large proportion of the overall inflation in consensus transcript number from this dataset may result from alternative splice isoforms or sequencing of incompletely processed mRNA molecules. It should also be noted that this analysis will confound lineage‐specific gene duplications with divergent haplotypes and splice isoforms; however, this bias will be consistent across all three species.

**Table 3:** Alignment rates of consensus transcripts generated from each of the three target species to the sorghum gene space

Species	Sorghum gene space coverage	Sorghum syntenic genes space coverage	Transcript alignment rate
*Hymenachne amplexicaulis*	11,485 genes/34,211 genes (33.5%)	6,402 transcripts/11,800 genes (54.2%)	115,361 transcripts/193,422 transcripts (59.6%)
*Chasmanthium laxum*	13,446 genes/34,211 genes (39.3%)	7,418 transcripts/11,800 genes (62.8%)	125,357 transcripts/164,640 transcripts (76.1%)
*Dichanthelium oligosanthes*	14,159 genes/34,211 genes (41.3%)	7,760 transcripts/11,800 genes (65.7%)	171,465 transcripts/190,632 transcripts (89.9%)

For each sorghum gene which aligned to two or more consensus transcripts from the same target species, a single representative transcript was selected for further downstream analysis (see Methods). Between 11,485 and 14,159 sorghum genes had a corresponding representative transcript in a given target species (Table 3). Here, we were (a) using a single library constructed per species, rather than multiple libraries constructed using different size fractions; (b) using RNA from a single tissue rather than pooled RNA from multiple tissue types; and (c) conducting comparisons between more distantly related species. However, the total proportion of sorghum genes represented in each transcriptome dataset was not substantially lower than the 14,401 *T. dactyloides*‐maize gene pairs identified in a previous study which implemented all of these best practices (Yan et al., 2019). This may in part be explained by both sequencing and library preparation improvements between the RSII and Sequel iterations of this sequencing technology.

**Figure 2:**
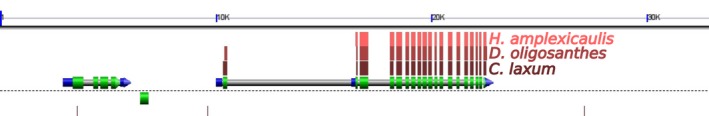
Transcript coverage of the C_4_ PPDK gene in Sorghum bicolor Sobic.009G132900 in each of the three species texted. Red‐brown boxes represent regions of similar sequence identified by BLASTN between the sorghum genome and consensus transcript sequences retrieved from *Hymenachne amplexicaulis*, *Dichanthelium oligosanthes*, *Chasmanthium laxum* (from top most to bottom most). The bottom track indicates the annotated gene structure, with intronic sequence indicated in gray and exonic sequence indicated in either blue (5' or 3' untranslated regions) or green (coding sequence). Top y‐axis indicates scale of the displayed genomic region in kilobases

Manual curation was used to access the coverage and quality of sequences retrieved from these three C_3_ photosynthesis‐utilizing PACMAD species for five genes known to be involved in C_4_ photosynthesis: PPDK, PEPC, NADP‐MDH, NAD‐ME, and DCT2 in C_4_ photosynthesis‐utilizing PACMAD species. In four cases, the representative transcript identified from each of the three target species spanned every annotated codon in sorghum. The one exception was PPDK where the representative transcript identified for *H. amplexicaulis* lacked the first annotated exon of the annotated gene model in sorghum (Figure 2). Multiple isoforms of the PPDK gene have been described in both maize and sorghum, with the shorter isoform, lacking the same exon absent in *H. amplexicaulis* (Sheen, 1991; Wang et al., 2009). This shorter isoform lacks the chloroplast transit peptide and encodes cytosolic PPDK protein not thought to be associated with C_4_ photosynthesis (Glackin & Grula, 1990; Sheen, 1991; Wang et al., 2009). However, these results would also be consistent with sequencing of an incomplete transcript from *H. amplexicaulis* with break point occurring at the same location as the 3′ junction of the first exon.

Phylogenetic consistency was assessed using a small subset of genes with high confidence syntenic orthologs identified in species with published reference genomes and representative transcripts identified in each of the three target species. A subset of PACMAD species with sequenced reference genomes was included in these analyses. Excluded species included those with fragmented genome assemblies at the time of these analyses as well as many, but not all, species with independent whole genome duplications (i.e., *Panicum virgatum*, *Miscanthus x giganteus* and *Eleusine coracana*) as these increased the complexity of downstream analyses. Two well‐characterized C_4_‐related proteins PPDK and PEPC were evaluated in detail. PPDK is a member of a small gene family, while genes encoding PEPC proteins are more numerous in many grass species. Copies of each of these two genes in sorghum previously identified as involved in the C_4_ cycle were identified from the literature Christin et al. (2007) and Wang et al. (2009). Phylogenies constructed using known gene copies from species with published reference genomes and IsoSeq transcripts of both PPDK and PEPC clustered all C_4_ gene copies together (Figure S3). A total of 11,800 genes were identified at syntenic orthologous locations across the genomes of rice, brachypodium, teff, Oropetium, pearl millet, foxtail millet, sorghum, and maize. Of these in 2,774 cases, no representative transcripts were retrieved from *C. laxum*, *D. oligosanthes*, or *H. amplexicaulis*. These cases likely represent conserved genes that are not expressed in developing photosynthetic tissue. In 1,611 cases, a representative transcript was identified in only one of the three target species, and in 2,276 cases, representative transcripts were identified in two of the three target species. In the remaining 5,139 cases, representative transcripts were retrieved for all three target species. The complete lists of each of these sets of conserved syntenic genes and corresponding transcripts from 0, 1, 2, or 3 of the target species are provided as part of Supporting Information.

One potential concern in using transcriptome data from species utilizing C_3_ photosynthesis to provide sequence data for comparative genetic and evolutionary analyses of C_4_ is that enzymes involved in the C_4_ cycle will likely different functions unrelated to photosynthesis in C_3_ plants (Aubry, Brown, & Hibberd, 2011), and therefore may not be expressed in photosynthetic tissue and hence be missing from datasets derived from sequencing cDNAs. Of 31 core C_4_ genes enumerated in (Huang et al., 2016), 20 were part of the set of 11,800 sorghum genes with conserved syntenic orthologs identified in each of the tested grass species with a published reference genome. Hence, these genes are almost certainly present within the genomes of *C. laxum*, *D. oligosanthes*, and *H. amplexicaulis* as well, whether or not they were expressed to sufficient levels to be detected in this analysis. Of these 20 syntenically conserved C_4_‐related genes, sequence data were obtained from all three target C_3_‐utilizing panicoid species in 16 cases. In the remaining four cases—DCT4c, GLR, NADP‐ME, and SCL—no putatively orthologous transcript was identified in any of the three species. There were no cases where a syntenically conserved gene linked to C_4_ photosynthesis was detected in some, but not all, of the three C_3_‐utilizing species evaluated.

From the list containing a total of 5,139 conserved orthologous gene groups present in all species, 231 were discarded for one of several reasons, listed from most common to least common. (a) In 113 cases, the CDS sequence for the *O. thomaeum* genome included one or more in‐frame stop codons. (b) In 61 cases in at least one species represented by IsoSeq data, no stop codon was present in any of the 6 possible open reading frames, indicating either a sequencing error or incomplete 3 prime coverage. (c) In 56 cases, a syntenic orthologous gene present in version 2.1 of the *B. distachyon* genome had been removed or renamed in version 3.1 of the *B. distachyon* genome. (d) One *O. thomaeum *de novo predicted gene region was not present in the CDS data.

The remaining set of 4,908 conserved orthologous gene groups were used to generate protein‐guided codon multiple sequence alignments (see Methods). A subset of these alignments containing at least 900 nucleotides (300 codons) alignment scored as "high quality" by GBlocks was employed to construct individual gene‐level trees (Figure S4). In total, 733 trees representing 267 putatively orthologous gene groups were constructed. Multiple trees resulted from retained duplicate gene pairs resulting from lineage‐specific whole genome duplications in maize and teff. Each duplication had the potential to create a retained syntenic gene pair which were each co‐orthologous to single gene copies in other grass species within the analysis. In order to maintain a consistent number of final nodes, when a retained gene pair was observed in one or both species, multiple sampled trees were generated (see Methods). A modest bias toward overrepresentation of retained—rather than fractionated—genes was observed in the set of genes which were represented in the transcriptome assemblies from all three target species: 37% (1,843/4,908) of maize genes in this set were retained as duplicate pairs versus 30% of all syntenic maize genes, and 100% of teff genes in this set were retained as duplicate pairs versus 91% of all syntenic teff genes. Dating multiple whole genome duplication events back to the Rho event in grasses is complex (McKain et al., 2016). Therefore, the lack of consideration of these events back to the Rho event could cause a limitation/ambiguity in the gene trees. The rice and brachypodium clade represented a known outgroup as these two species belong to the BOP clade which diverged from the PACMAD clade of grasses early in the evolution of this family (GPWG II, 2012). In 49 cases, RAxML was unable to place the rice‐brachypodium clade as an outgroup suggested broader issues with orthology assignment, correct ORF identification, or alignment. These trees were not included in downstream analyses.

**Figure 3:**
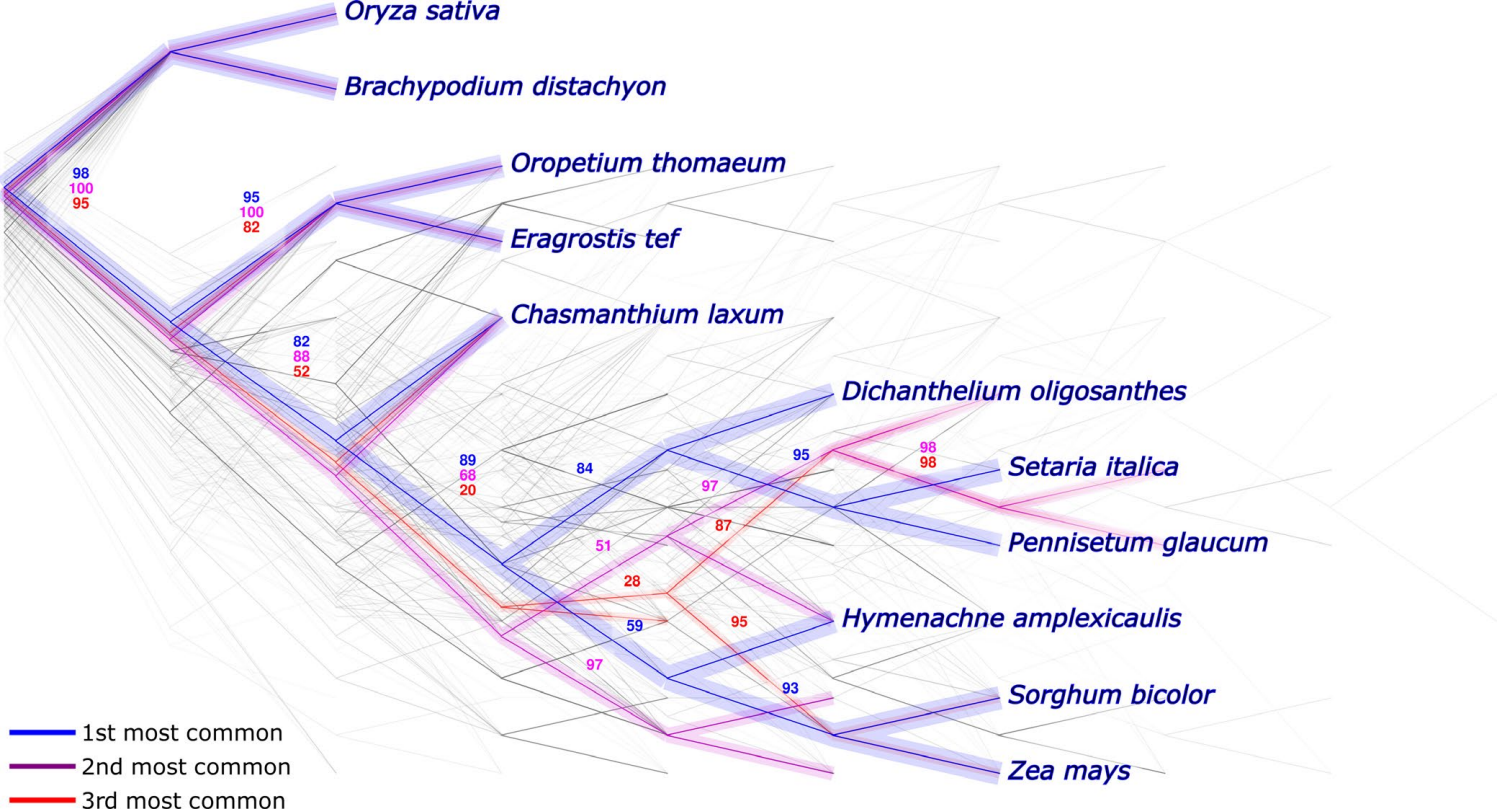
Seven hundred distinct phylogenetic trees calculated from separate multiple sequence alignments of 267 putatively orthologous gene groups with large regions of alignment scored as high quality. Blue indicates the most commonly observed topology (291 trees (42.5% of the total), purple and red indicate the second (43 trees (6.2%) and third most commonly observed topologies (28 trees (4%)), respectively. Numerical labels of branches for each topology indicate average bootstrap support from separately calculated bootstrap trees for 100 randomly selected gene groups, considering data from those gene trees consistent with that particular topology

Among the 684 remaining gene trees, 291 (42.5%) produced a single topology consistent with the prior literature on the relationship of these species (Figure 3). The second and third most common topologies were each represented by less than 7% of all calculated trees, 43 and 28 cases, respectively. The second and third most common topologies differed from prior published phylogenies regarding the placement *H. amplexicaulis*. In the second most common topology, *H. amplexicaulis* was placed sister to all other panicoid grass species other than *C. laxum*. In the third most common topology, *H. amplexicaulis* was placed sister to the Paniceae. Parallel analysis was conducted using all 4,908 conserved orthologous gene groups, including many cases with substantially shorter regions of high‐quality multiple sequence alignment. The pattern of trees recovered was largely consistent with those in Figure 3. In the "all genes" analysis, the same first and second most common topologies were retrieved as in the long alignment only analysis. The third most common in the "all genes" topology places *C. laxum* as sister to the combined Chloridoideae and Panicoideae (Figure S5).

A bootstrap analysis was performed, and values were retrieved for each of the three most common topologies. In the analysis, 100 randomly trees were chosen to perform the bootstrap analysis (see Methods). The most common topology of the 100 trees was the same as the one observed in Figure 3, appearing seven times. Both second and third common topologies in Figure 3 only appeared once. The bootstrap values observed for the most common topology are the average of all seven bootstrap values of each branch (Figure S6). The bootstrap values of the internal branches in the second and third most common topologies are substantially smaller than the values obtained in the most common topology. Overall, the most common topology exhibits stronger bootstrap support values compared to the other two topologies.

## CONFLICT OF INTEREST

The authors declare that they have no competing interests.

## AUTHOR'S CONTRIBUTIONS

DSC and JSC wrote the paper and designed the experiments, and DSC generated and analyzed the transcriptome data. All authors have reviewed and approved the manuscript.

## Data Availability

Raw sequence data for *C. laxum*, *H. amplexicaulis*, and *D. oligosanthes* have been deposited in the NCBI SRA under accessions numbers: SRR7632721 (*C. laxum*), SRR7632716 (*H. amplexicaulis*), and SRR9603193 (*D. oligosanthes*). Processed consensus transcript sequences generated for each of these three species have been deposited in Zenodo https://doi.org/10.5281/zenodo.3253206. Syntenic gene sets and putatively orthologous relationships are provided as Supporting Information.
