# Supplementary figures and images for: IsoSeq transcriptome assembly of C3 panicoid grasses provides tools to study evolutionary change in the Panicoideae
Source: Plant Direct. 2020 Feb 28;4(2):e00203. doi: 10.1002/pld3.203 (PMC7047018; doi:10.1002/pld3.203)

**A**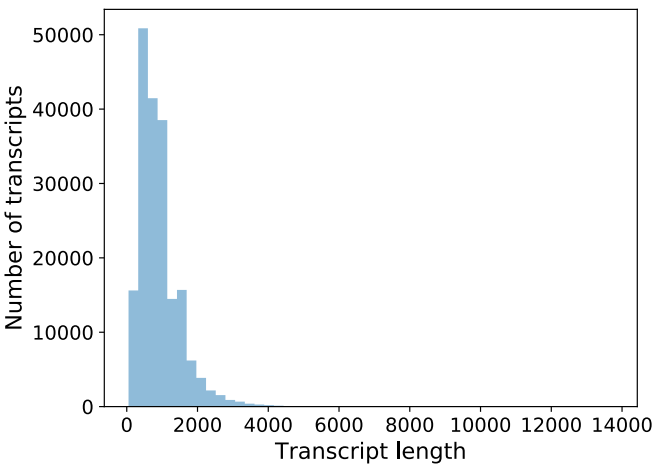**B**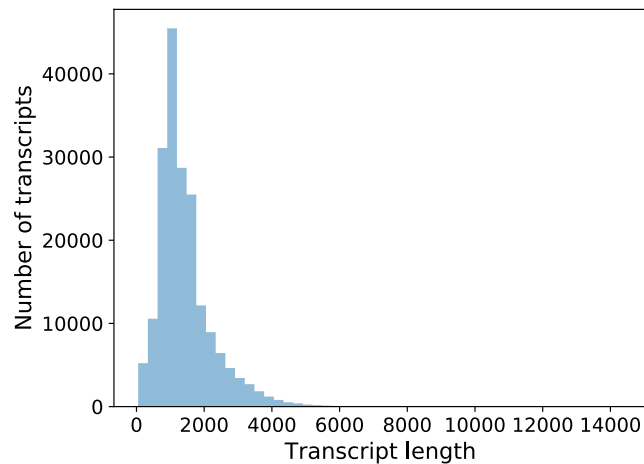**C**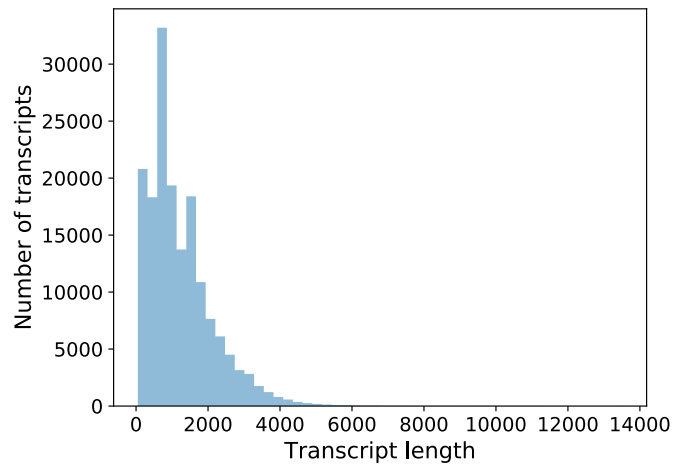

Supplement: Supplementary file 1 [file PLD3-4-e00203-s001.pdf]

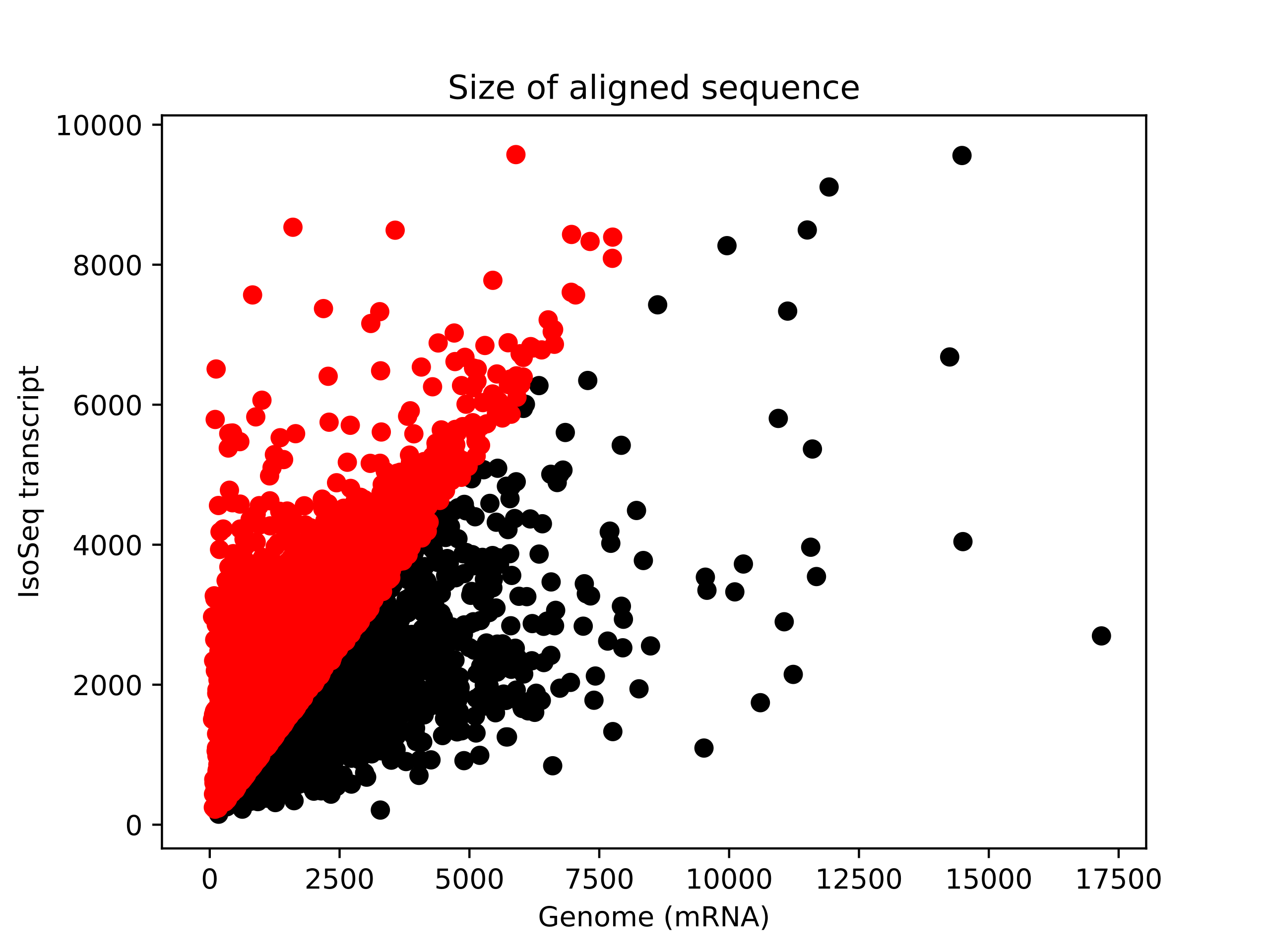

Supplement: Supplementary file 2 [file PLD3-4-e00203-s002.png]

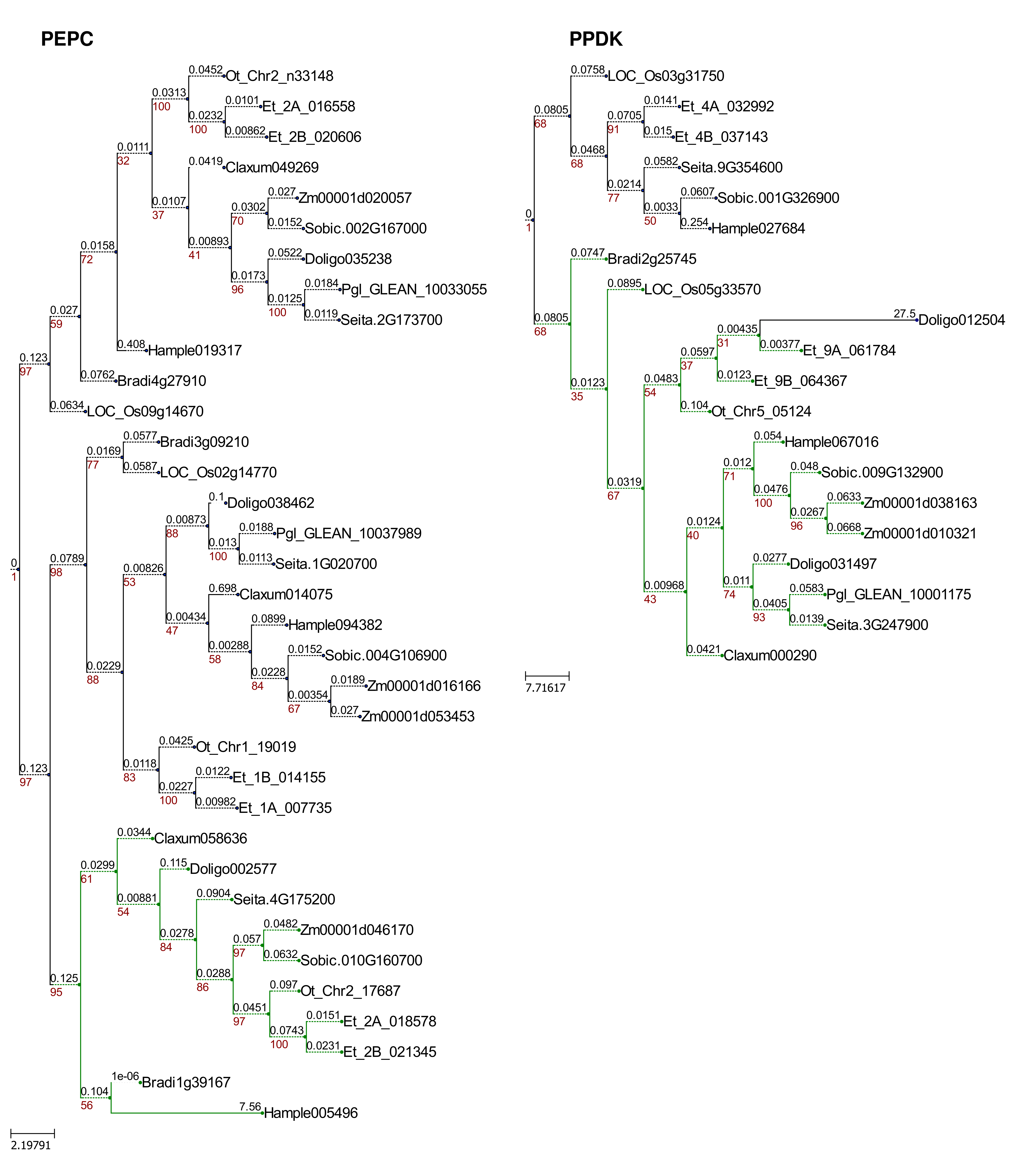

Supplement: Supplementary file 3 [file PLD3-4-e00203-s003.png]

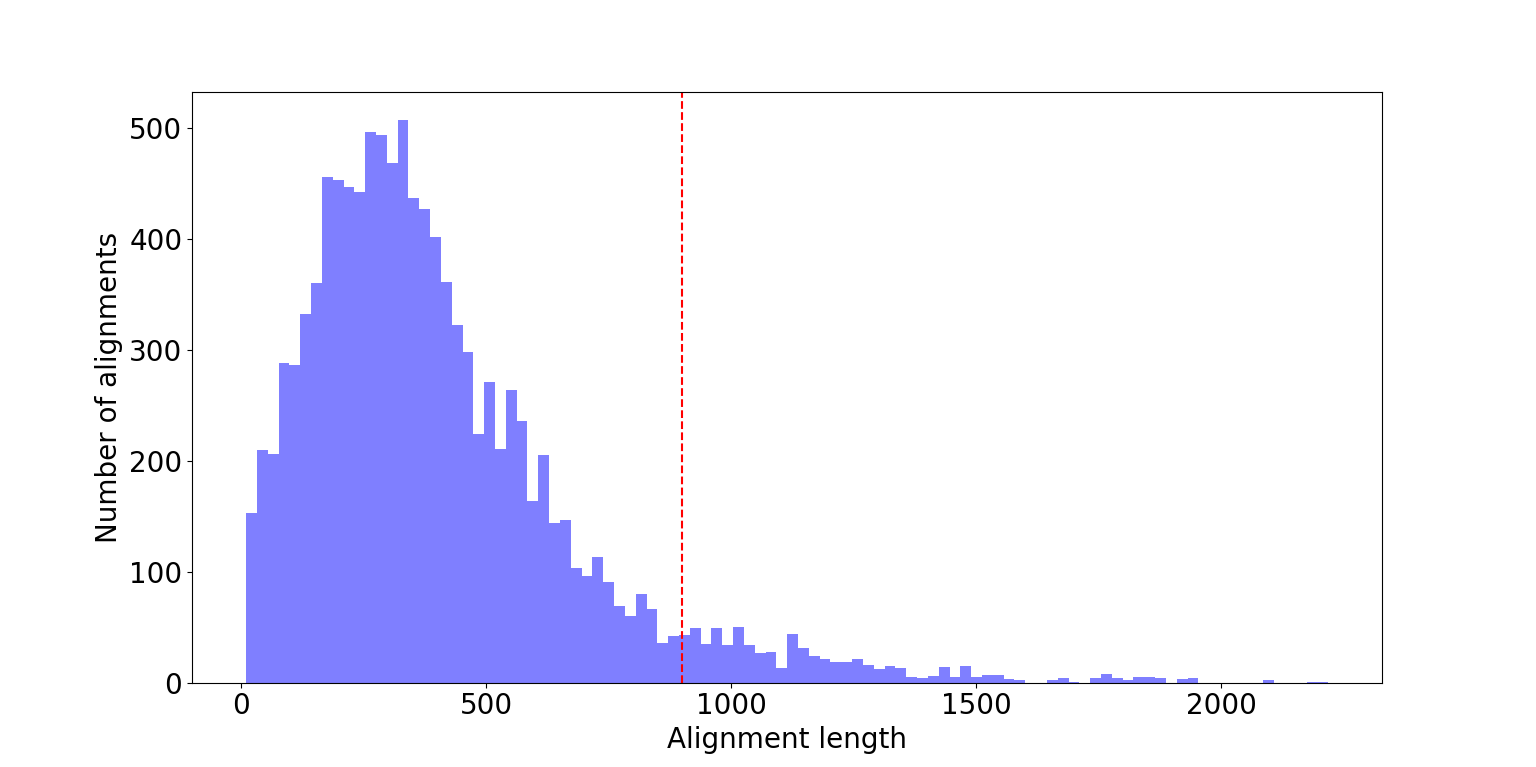

Supplement: Supplementary file 4 [file PLD3-4-e00203-s004.png]

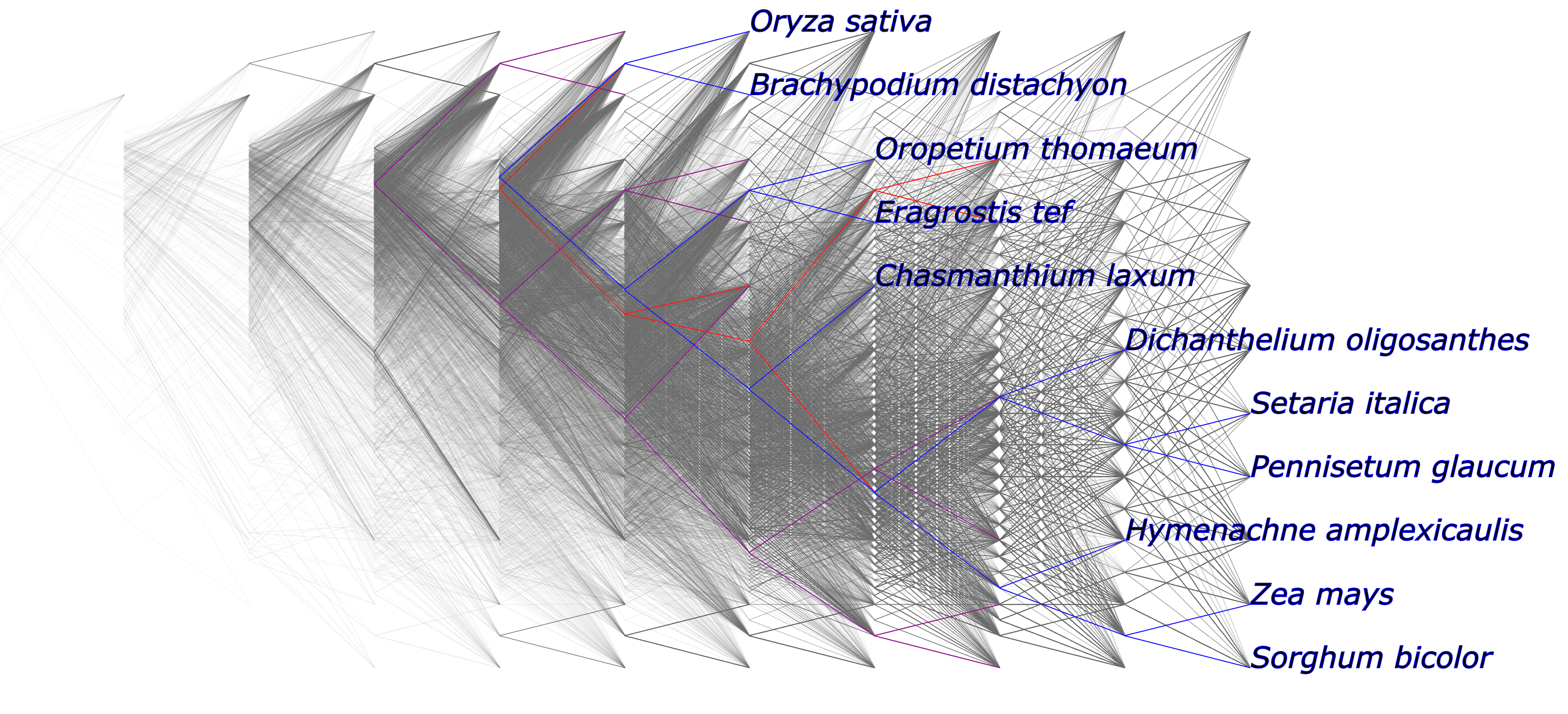

Supplement: Supplementary file 5 [file PLD3-4-e00203-s005.png]

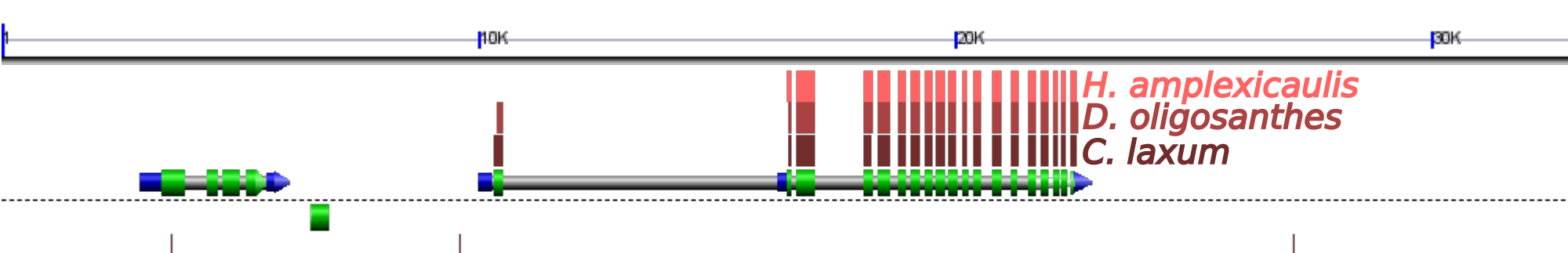

Supplement: Supplementary file 6 [file PLD3-4-e00203-s006.pdf]
