# Supplementary material for: IsoSeq transcriptome assembly of C3 panicoid grasses provides tools to study evolutionary change in the Panicoideae
Source: Plant Direct. 2020 Feb 28;4(2):e00203. doi: 10.1002/pld3.203 (PMC7047018; doi:10.1002/pld3.203)
Supplement: Supplementary file 8 [file PLD3-4-e00203-s008.pdf]

Reviewer #1:

I appreciate that the authors have made a number of positive changes to the manuscript. I think that it is improved as a result. I have a few minor things below. For the second point, I am expressing the possibility that there could be more conflict in these trees than suggested by using only the best tree. This could change the interpretation of the phylogeny, though my suspicion is that the relationships of these sampled taxa is fairly well known.

We thank the reviewer for the feedback. The reviewer is correct, the relationship of these taxa is well known.

1) I appreciate the addition of more information regarding the quality of the RNA that went into IsoSeq. However, 260/280 values only reflect purity of the sample. They do not reflect degradation, which would be important for IsoSeq.

We understand where the reviewer is coming from, but unfortunately this is information that simply was not collected for the RNA samples at the time of sequencing. We can (and now have) noted that this information would be useful but was not collected. Beyond that we simply do not have any option to address the reviewer's concern open to us.

2) I understand where the authors are coming from with the bootstrap-less phylogeny reconstruction, but it is important to consider that best trees might be minimally more likelihood than other topologies. Similar analyses using Densitree on bootstrapped trees might demonstrate conflicting resolution in gene trees themselves. If a Densitree analysis of bootstrap trees for all genes was done, it might alter the relative percentages of most common topologies. It is possible that the conflict see in some of these trees may be more prevalent but masked by using only the best trees.

We agree with the reviewer that if we were focusing on performing phylogenetic analyses to investigate a poorly resolved evolutionary relationship among species this change could have a significant impact on the results. However, it would be unfeasible to perform bootstrap analyses for all trees in a timely manner. To address this limitation we have randomly chosen 100 trees (out of the total 11,371 trees generated) to run 100 bootstraps. Also, we performed bootstrap analyses on the PPDK and PEPC trees (1000 replicates). Text was added to methods to explain how the bootstrap analysis was performed.

After bootstrapping, polytomies were much more common than in the original trees indicating the low resolving power of individual gene trees rather than looking at patterns observed across many distinct trees generated from the alignment distinct orthologous gene groups. However, among the 100 genes tested, we still identified 7, 1, and 1 genes where the topology of the bootstrapped tree agreed with the most common, second most common, and third most common trees identified in the gene by gene analyses. Bootstrap support for individual branches on each of the three most common trees in in Figure 3 was estimated separately using average values from those genes exhibiting that same topology. As shown in our revised Figure 3, most branches have relatively strong bootstrap support in individual gene trees, however the branches where the second and third most common topologies different from the most common topology tend to have low bootstrap support in individual gene trees.

Now we have updated figure 3 with the bootstrap values observed for all three topologies. Because the most common topology had 7 trees and 7 bootstrap values for each branch, the values seen for the most common topology show the average bootstrap values. We included another supplemental figure (Figure S6) that shows all 7 bootstrap values for this tree. We also added text to the discussion about the bootstrap values obtained and updated figure 3 to show the bootstrap values of the 3 most common topologies.

The results of PEPC and PPDK trees show that some branches indeed show low bootstrap support and indicate conflicts in the trees, as expected by the reviewer. As our goal was to

show the overall quality of the Iso-Seq data and its suitability to phylogenetic studies of C3 and C4 grasses, we did not perform deeper analyses about the evolution of the group, as it is well resolved in a more robust phylogeny (GPWG II, 2012 doi: 10.1111/j.1469-8137.2011.03972.x).

Line 290: Oropetium should be italicized, unless it is used as a common name for the taxon.

Oropetium is used as a common name for the species.

Fig. S3: Include in the figure legend information about the type of tree (e.g. maximum likelihood). What do the number indicated on the branches? Branch lengths? Branch lengths represented on the tree are not usually written.

We apologize for the mislabeling. It was a maximum-likelihood tree and the numbers represent branch lengths. Figure legend was updated.

For this tree, the lab of bootstrap values is a negative. I would suggest running at least these trees with bootstrap values.
We agree with the reviewer and have performed the bootstrap analyses, as mentioned in 2). Figure S3 now shows both the branch lengths and bootstrap values.

Fig. S5: Names on tree tips need to be consistent and recognizable. Why not have names mirror Fig 1A or 3?

We have updated Figure S5.

Reviewer #2:

The authors have addressed my comments and concerns in their revision.
